# A comparison of the *Caulobacter* NA1000 and K31 genomes reveals extensive genome rearrangements and differences in metabolic potential

**DOI:** 10.1098/rsob.140128

**Published:** 2014-10-01

**Authors:** Kurt Ash, Theta Brown, Tynetta Watford, LaTia E. Scott, Craig Stephens, Bert Ely

**Affiliations:** 1 Department of Biological Sciences, University of South Carolina, Columbia, SC 29208, USA; 2 Biology Department, Santa Clara University, Santa Clara, CA 95053, USA

**Keywords:** *Caulobacter*, plasmids, genome rearrangements, gene insertions, inversions

## Abstract

The genus *Caulobacter* is found in a variety of habitats and is known for its ability to thrive in low-nutrient conditions. K31 is a novel *Caulobacter* isolate that has the ability to tolerate copper and chlorophenols, and can grow at 4°C with a doubling time of 40 h. K31 contains a 5.5 Mb chromosome that codes for more than 5500 proteins and two large plasmids (234 and 178 kb) that code for 438 additional proteins. A comparison of the K31 and the *Caulobacter crescentus* NA1000 genomes revealed extensive rearrangements of gene order, suggesting that the genomes had been randomly scrambled. However, a careful analysis revealed that the distance from the origin of replication was conserved for the majority of the genes and that many of the rearrangements involved inversions that included the origin of replication. On a finer scale, numerous small indels were observed. K31 proteins involved in essential functions shared 80–95% amino acid sequence identity with their *C. crescentus* homologues, while other homologue pairs tended to have lower levels of identity. In addition, the K31 chromosome contains more than 1600 genes with no homologue in NA1000.

## 2. Introduction

Stalked bacteria from the genus *Caulobacter* are ubiquitous inhabitants of aquatic ecosystems, particularly in oligotrophic habitats in which biologically available concentrations of organic matter and other nutrients are very low. To gain further insight into the physiological diversity of the genus and the evolution of *Caulobacter* genomes, we compared the genomic DNA sequences of two *Caulobacter* species isolated from different habitats: CB15 (also known as NA1000), isolated from a surface freshwater site [1]; and K31, a groundwater isolate of particular interest for its ability to tolerate and degrade chlorophenols [2]. The genomic DNA sequence of *Caulobacter crescentus* CB15 [3,4], along with a corrected annotation [5], is available in the GenBank database (http://www.ncbi.nlm.nih.gov).

Caulobacters are Gram-negative bacteria that are of interest to microbiologists for several reasons. Perhaps foremost, their unique dimorphic life cycle includes motile and non-motile (stalked) cell types, produced during an obligatory cell cycle progression [6]. The motile cell is an immature cell, which must lose its flagellum and differentiate into a stalked cell before it can replicate its chromosome and divide. This dimorphic *Caulobacter* life cycle may have evolved to allow exploitation of distinct strategies for nutrient acquisition. In addition to their adherent stalked stage, the chemotactic ‘swarmer’ cells can actively seek nutritionally optimal microhabitats. Swarmer cells are analogous to the G1 phase of the eukaryotic cell cycle in which DNA replication is blocked. Also, stalked cells do not initiate the next round of chromosome replication until after cell division has been completed. Therefore, caulobacters do not initiate overlapping rounds of chromosome replication.

The ease with which swarmer cells of *C. crescentus* can be isolated in high yield allows investigators to prepare cultures that are synchronous with respect to the cell cycle. In the presence of adequate nutrients, progression through the *Caulobacter* cell cycle is controlled by an internal clock that eventually directs the swarmer to eject its flagellum and progress to the sessile, stalked stage of the life cycle [6]. This maturation is accompanied by the initiation of DNA replication (analogous to S phase). Subsequent developmental events (flagellum and pili synthesis, cell division) depend on progression and completion of chromosome replication. Aided by the availability of the genome sequence and a suite of genetic techniques [3,4,7], excellent progress has been made in outlining the genetic regulatory network and signal transduction pathway controlling the *C. crescentus* cell cycle [8–10].

Less effort has gone into applying genomics to understanding the environmental biology of caulobacters, which form tightly adherent, stable biofilms on submerged surfaces [11,12]. Prokaryotes represent a vast reservoir of biomass interacting with the earth's water supplies and bear significant responsibility for the cycling of carbon, nitrogen and phosphorus in the biosphere. Members of the alpha subdivision of *Proteobacteria*, including the genus *Caulobacter*, are ubiquitous components of the microbiota of virtually every habitat examined. This group is especially prominent in oligotrophic habitats, which include vast tracts of open ocean and subsurface aquifers. Heterotrophic microbes with high affinities for organic compounds complete the mineralization of carbon in such habitats [13]. However, our understanding of the molecular and genetic adaptations of microbes for oligotrophy is very limited. The genomic DNA sequence of *C. crescentus* suggests a variety of possible adaptations, including an extraordinarily large set of membrane transport systems [3].

An analysis of ribosomal RNA sequences and other data has shown that stalked bacteria previously assigned to the genus *Caulobacter* actually fall into two distinct branches comprising freshwater and marine genera [14,15]. The freshwater genera include *Caulobacter* and *Brevundimonas*. A comparison of the *Caulobacter* 16S rDNA sequences indicates two well-supported branches: one containing *C. segnis* and various *C. crescentus* isolates, and the other containing *Caulobacter* isolate FWC20 and *C. henricii* [15,16]. Although the genomes of 37 *Caulobacter* isolates are listed in the IMG database (img.jgi.doe.gov), only four are listed as complete, and two of these (CB15 and NA1000) are different versions of the same isolate. In addition, the ‘finished’ genome of *C. segnis* contains numerous sequencing errors and the annotation needs to be corrected to include more than 100 genes that are not present in the publically available version of the genome [17]. The other finished genome, described in this study, belongs to K31, a groundwater isolate that is most closely related to the *C. henricii* lineage, based on a partial 16S rRNA sequence [2]. K31 was poorly characterized, but its abilities to adapt to a low-oxygen groundwater habitat and to tolerate and degrade chlorophenols make it relevant to groundwater bioremediation [2]. As the K31 genome is from a different branch of the *Caulobacter* genus relative to the well-studied *C. crescentus* CB15/NA1000 genome, we chose to compare the two genomes to provide a representation of the genetic diversity of the genus. We also provide a preliminary characterization of the growth and metabolism of the K31 strain that illustrates some of the adaptation this strain has made to life in a cooler subterranean habitat.

## 3. Material and methods

### 3.1. Media and growth conditions

The *Caulobacter* K31 strain was obtained from Minna Mannisto (Arctic Microbiology Research Consortium, Ravoniemi Research Station, Finland), the investigator responsible for its original isolation. When K31 was first isolated, it was grown in PYGV medium, which contains equal amounts of peptone, yeast extract and glucose (0.025% w/v) plus a vitamin solution and 1.5% agar [2]. To determine the optimal growth conditions for K31, we varied the components of the growth medium. We found that riboflavin stimulates growth on minimal media plates (M2G) [18]. Previous experiments with *C. crescentus* strain CB15 showed that it grew better in PYE (0.2% Bacto peptone, 0.1% yeast extract, 0.75 mM MgSO_4_, 0.5 mM CaCl_2_) [18] in the presence of both glucose and glutamate than with either addition alone (Ely laboratory 1977, unpublished data). In similar experiments with K31, growth with PYE plus glucose caused the pH of the culture fluid to go down and growth with PYE plus glutamate caused the pH to go up. When glucose and glutamate were added together, the pH stayed between 7 and 8. Thus, the growth conditions for optimal yield of K31 are PYE supplemented with 10 mM glucose, 30 mM monosodium l-glutamate and 10 mM riboflavin (PYEGGR). The growth rate is essentially the same in PYE with no additions; however, the final yield was increased about threefold when the additives were present. In contrast to NA1000, which grows at maximum growth rate at 33–35°C [18], K31 failed to grow at 35°C. At 30°C, K31 had a doubling time of 160 min, significantly slower than the 110 min doubling time of NA1000 at 30°C. Our experiments also revealed the ability for K31 to grow at 4°C with a doubling time of about 40 h. The ability to grow at low temperatures is consistent with the fact that K31 was isolated from cold groundwater [2].

To determine growth in the presence of copper, a 60 mg ml^−1^ of copper sulfate stock solution was diluted to provide a range of concentrations, and 100 µl of each dilution was added to 10 ml of a K31 or NA1000 culture in PYEGGR. After growth at 30°C for 48 h, the optical density of each culture was measured using a Klett–Summerson colorimeter.

### 3.2. Genome sequence determination and annotation

Genomic DNA from *Caulobacter* strain K31 was isolated from a saturated culture using the Qiagen Genomic-tip system, following the manufacturer's protocol. Subsequent library construction and nucleotide sequencing were carried out at the Joint Genome Institute (JGI) of the US Department of Energy. Genomic DNA was sheared to generate the appropriate DNA size fragments (3, 8 and 40 kb) and was verified by gel electrophoresis. The sheared DNA was repaired to produce blunt-end fragments and was subsequently ligated into a series of bacterial plasmids: pUC18 for 3 kb inserts, pMCL200 for 8 kb inserts and pCC1Fos for 40 kb inserts. Plasmids were transformed by electroporation into *Escherichia coli* and plated on selective media, then picked and archived. To generate sequencing templates, plasmid DNA was isolated from cells and subjected to rolling-circle amplification (RCA). RCA products were subsequently used for Sanger sequencing and capillary gel electrophoresis. Assembly of the genome entailed alignment of sequences from all three libraries using Phrap. After quality assessment of the draft assembly, the sequence was automatically annotated by JGI using their standard annotation system. The GenBank[/EMBL/DDBJ] accession numbers for the genome sequence of *Caulobacter* strain K31 are CP000927, CP000928 and CP00029.

### 3.3. Genome comparisons

For genome comparisons, the K31 genome was compared to the *C. crescentus* NA1000 genome, which is thought to be most closely related to the original isolate of the CB15 laboratory strain [4]. The two genomes were aligned and compared using the program ProgressiveMauve [19]. Large inversions and translocation were estimated by a manual count of the aligned genomes. Annotations of the two genomes were viewed and analysed in Artemis [20]. A BLAST comparison to identify homologous genes in the NA1000 and K31 genomes was performed using the protein-coding sequences of all the CDS regions of the chromosomes for each bacterium. The comparisons were conducted using the BlastStation 2 Windows-based software (http://www.blaststation.com). BLAST matches with an e-value that was less than ×10^−5^ were considered significant.

## 4. Results and discussion

### 4.1. Genome overview

**Figure 1.**
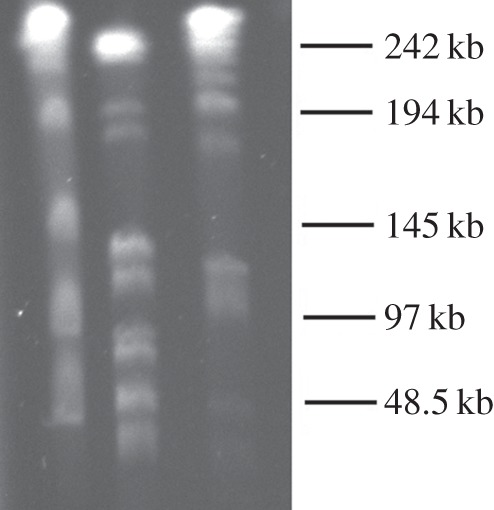
Pulse field gel electrophoresis of *Ase*I and *Spe*I-digested DNA. Lane 1, lambda size ladder with sizes indicated to the right of the gel. Lane 2, *Ase*I digest of K31 DNA. Lane 3, *Spe*I digest of K31 DNA.

The *Caulobacter* K31 genome consists of a 5 477 872 base pair (bp) chromosome and two plasmids. The larger plasmid contains 233 649 bp, and the smaller contains 177 878 bp. The K31 chromosome has a 68.1% GC content and contains 5061 genes (table 1). The accuracy of the genome assembly was confirmed by pulse field gel electrophoresis of *Spe*I-digested K31 genomic DNA (figure 1). There was a one to one correspondence between the bands observed on the gel and those predicted from the nucleotide sequence of the main chromosome. However, one extra 233 kbp band was present in the gel that corresponded to the predicted band from cleavage of pCAUL01 DNA at the single *Spe*I site predicted for pCAUL01. The pCaul02 plasmid is also predicted to have a single *Spe*I site that would generate a 178 kbp fragment that would co-migrate with a restriction fragment from the main chromosome. Therefore, to verify the presence of the pCAUL02 plasmid, we digested total K31 DNA with *Sna*BI, which also cut pCAUL02 DNA once. In this case, the resulting 178 kbp fragment was clearly visible on the PFGE gel because digestion of the main chromosome does not produce any fragments that are close in size. Thus, we were able to verify the presence of both plasmids using the PFGE analyses.

**Table 1.** A comparison of *Caulobacter* strains NA1000 and K31.

*Caulobacter* isolate	NA1000	K31
source	pond water	groundwater sample
cell shape	crescent	crescent
genome features		
base pairs	4.02 × 10^6^	5.48 × 10^6^
plasmids	0	2
G/C content	67.2%	67.4%
protein-coding genes	3876	5443
tRNA genes	51	49
rRNA genes	6	6
5s	2	2
16s	2	2
23s	2	2
flagellar genes	43	44
phage genes	15	42
transposases	40	61
integrases	4	33
recombinases	2	7
pseudogenes	1	17

The K31 chromosome contains more than 1900 genes that do not appear to have a homologue in NA1000. Conversely, the NA1000 chromosome contains more than 200 genes that do not appear to have a homologue in K31. As with the NA1000 chromosome, the K31 chromosome has two identical ribosomal RNA operons. However, the K31 rRNA operons are adjacent to each other, whereas the NA1000 rRNA operons are separated by more than 90 000 bp. In contrast to the 97% sequence identity of the K31 and NA1000 rRNAs, the ribosomal internal transcribed spacer (ITS) regions of the two species are only 80% identical. Thus, considerable divergence has occurred between the K31 and NA1000 ITS regions even though the two copies of the ITS region are identical within each species.

When the COG categories of the K31 genes were compared with those of NA1000, the K31 gene counts were higher in most categories due to its larger genome, but genome percentages were similar for most categories (table 2). Exceptions were translation, motility and nucleotide transport, and metabolism categories where additional genes are not likely to serve a useful purpose. Examples where K31 has more genes include 14 additional sigma factors, more than 150 additional regulatory proteins, 19 additional response regulators, 32 additional TonB-like receptors, 22 additional major facilitator transporters, nine additional alcohol dehydrogenases, four additional ferredoxins and four additional cytochrome *c* class 1 genes. All of these genes code for proteins that are more similar to proteins produced in a variety of other bacteria than to any protein produced by NA1000. It remains to be determined whether NA1000 has lost these genes or K31 has gained these genes from other sources by horizontal gene transfer. It is more likely that some combination of the two processes has occurred. An important clue is that K31 has about the same number of transposase genes relative to the size of the genome as NA1000 does, but it only shares about one-third of them with NA1000. This comparison suggests that most of these transposases have been introduced from other species of bacteria and that horizontal gene transfer has played a major role in generating the differences in gene composition observed between these two species.

**Table 2.** K31 and NA1000 gene counts by COG category. Source: img.jgi.doe.gov.

COG category	K31 gene count	% of total *n* = 4109	NA1000 gene count	% of total *n* = 3037
amino acid transport and metabolism	260	6.33	222	7.31
carbohydrate transport and metabolism	219	5.33	157	5.17
cell cycle control, cell division and chromosome partitioning	33	0.80	23	0.76
cell motility	71	1.73	66	2.17
cell wall/membrane/envelope biogenesis	244	5.94	195	6.42
chromatin structure and dynamics	3	0.07	2	0.07
coenzyme transport and metabolism	132	3.21	114	3.75
defense mechanisms	71	1.73	45	1.48
energy production and conversion	231	5.62	165	5.43
function unknown	416	10.12	336	11.06
general function prediction only	483	11.75	344	11.33
inorganic ion transport and metabolism	220	5.35	153	5.04
intracellular trafficking, secretion and vesicular transport	137	3.33	83	2.73
lipid transport and metabolism	269	6.55	171	5.63
nucleotide transport and metabolism	75	1.83	71	2.34
post-translational modification, protein turnover and chaperones	155	3.77	130	4.28
replication, recombination and repair	222	5.40	127	4.18
secondary metabolites biosynthesis, transport and catabolism	163	3.97	100	3.29
signal transduction mechanisms	190	4.62	155	5.10
transcription	346	8.42	212	6.98
translation, ribosomal structure and biogenesis	169	4.11	166	5.47
not in COGs	1830	33.28	1178	29.95

One example of horizontal gene transfer may be the two K31 plasmids that code for an additional 438 proteins. The larger plasmid (pCAUL01) contains genes for conjugal transfer, and includes a collection of transporter genes and genes for the regulation of transcription. In contrast to the smaller plasmid and the main chromosome, the large plasmid contains only two adjacent transposase genes. It also contains a gene, Caul_5182, that codes for an integration host factor (IHF) protein that is 88–90% identical to the IHF proteins produced by two homologous chromosomal genes (CAUL_1806 and CAUL_2219). By contrast, NA1000 has a single homologous gene (CCNA_2416) that codes for the IHF protein. No other genes on pCAUL01 are homologous to any NA1000 gene. The smaller of the two plasmids (pCAUL02) contains genes for conjugal transfer (CAUL_5365–5374) that are present in the same order as those in pCAUL01 (CAUL_5213–5223), with amino acid identities ranging from 47 to 60%. Thus, the two sets of conjugal transfer genes appear to be distantly related. In addition, genes Caul5296–5310 comprise a region of pCAUL02 that codes for proteins responsible for the degradation of linear alkylbenzenesulfonate and is homologous to the corresponding genes in the alpha-proteobacterium *Parvibaculum lavamentivorans* (60–80% amino acid identity) with only one gene of the cluster falling below the percentage identity range shared by the rest of the genes. Thus, this set of degradative genes appears to have been acquired from a species of *Parvibaculum* or from some closely related genus. The pCAUL02 plasmid also appears to have experienced a number of transposition events as it contains 17 transposase genes, two of which have 95% amino acid identity to orfA and B of NA1000 IS511. However, they are located in two different regions of the plasmid and only 25% of the orfA gene is present. Only two of the other transposase genes code for proteins that are homologous to other NA1000 transposases. Two additional genes, Caul_5396 coding for a TonB-like receptor and Caul_5432 coding for an acyl carrier protein, are the only other pCAUL02 genes that code for proteins with more that 70% amino acid identity to a NA1000 protein. pCAUL02 also contains two anti-restriction genes that flank three phage integrase genes. These phage integrase genes are 78–91% identical to the corresponding genes from plasmid pACRY403 from *Acidiphilium cryptum* JF-5.

Phage genes are present in the main chromosome of K31 as well. A 12 kb region of phage genes is located adjacent to a gene that has 41% identity to *dnaJ* in both the K31 chromosome (beginning at nucleotide position 4220530) and the NA1000 chromosome (beginning at nucleotide position 3013149). As there is 66% nucleotide identity between the two regions and 76% nucleotide identity between the two *dnaJ*-like genes, these results suggest that the phage genes were present in the common ancestor of the two *Caulobacter* species. One difference between the two regions is a 400 base insertion in NA1000 (CCNA_2873) that disrupts a phage gene and codes for a bleomycin resistance protein that has 86% amino acid identity with a homologue found in *Cystobacter fuscus*. When the remaining homologous phage genes were compared, the amino acid identity ranged from 57% for a membrane protein of unknown function to 88% for the major capsid protein. For comparison, an 11 kb flagellar gene cluster that is required for motility has a 71% nucleotide identity when the two genomes are compared and the amino acid identity of the predicted proteins ranges from 59 to 93%. Thus, there appears to be a comparable level of nucleotide diversity and gene conservation between the phage and flagellar gene clusters. Sequence conservation in the flagellar gene cluster would be maintained by selection for motility, but it is not clear why the phage region should be conserved. Many of the phage genes code for structural proteins, but no phage particles have been observed in cultures of either strain.

Additional phage genes are found at four other locations in the K31 chromosome. One set of phage genes located in a 14 kb region beginning at nucleotide position 1648526 (CAUL_1559–1573) corresponds to a series of homologous genes found in the same order in *Bradyrhizobium* and *Chloroflexus*. A second set of phage genes located in a 15 kb region beginning at nucleotide position 1935379 and proceeding on the reverse strand (CAUL_1824–1812) corresponds to a series of homologous genes found in the same order in *Pelobacter*. A third region contains phage genes scattered throughout a 40 kb region with lower GC content (CAUL_3468–3507) and the fourth region contains three phage tail collar protein genes (CAUL_1097–1099) followed by nine transposase genes (CAUL_1101–1109). All four of these K31 phage regions have no homologues on the NA1000 chromosome suggesting that they were acquired by some type of horizontal gene transfer.

### 4.2. Codon usage

As the K31 chromosome contains a 68.1% genomic G+C content, the codons in the protein-coding regions should have a high G+C content, especially in the third codon position (GC3). In fact, 24 of the 25 most used codons contain either a G or a C in the third position. The overall GC3 percentage for K31 codons is 88.4%, slightly higher than the 86.9% observed in the NA1000 genome. The K31 codon usage table is similar to that of NA1000 as well, although K31 only has 49 tRNA genes compared with 51 in NA1000. Relative to NA1000, K31 has one copy instead of two copies of an asparagine tRNA, four copies instead of five copies of methionine tRNA, and three copies instead of two copies of an aspartate tRNA with a GTC anticodon. In addition, NA1000 has a leucine tRNA with a TAA anticodon that is not present in K31. The remaining tRNAs are present in both strains and have identical anticodons.

The GC content of pCAUL01 is 67.3% and the pattern of codon usage is similar to that of the K31 chromosome. By contrast, pCAUL02 has increased use of 29 of the 30 amino acid coding codons that end in A or U, consistent with its 64.3% GC content. The exceptional U-ending codon is UAU, where no increase is observed because UAU and UAC are used with almost equal frequency to code for tyrosine in both the plasmid and the chromosomal genes. This nearly equal use of the two tyrosine codons occurs in most *Alphaproteobacteria* with GC-rich genomes as well, and is an exception to the observation that G- and C-ending codons are used preferentially in these high GC chromosomes. In 19 of the 29 codons with increased use, the frequency of use is more than double that found in the main chromosome. Thus, a difference of less than 4% in average GC content corresponds to a major difference in the use of A- or U-ending codons, with third position GC content dropping from 88.2% in the main chromosome to 79% in pCaul02.

### 4.3. Genome rearrangements

**Figure 2.**
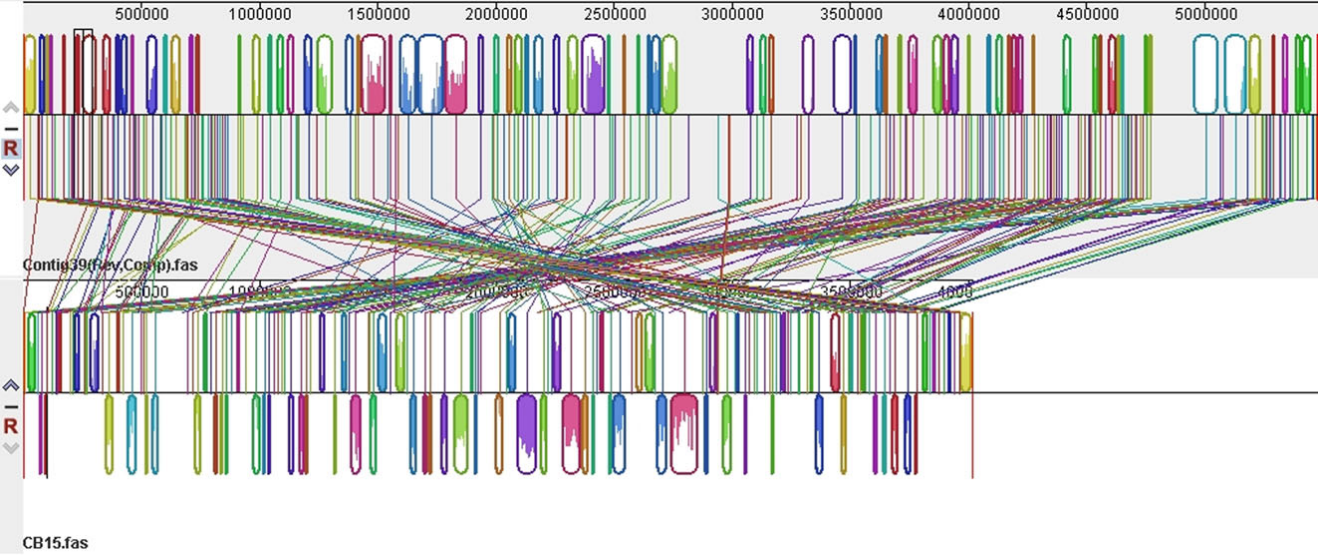
An alignment of the K31 chromosome with the *C. crescentus* NA1000 chromosome showing more than 60 inversions and 45 large translocations. Regions of contiguous homology have the same colour and are connected by a line of that colour.

When the genomes of closely related species are compared, they are usually collinear except where inversions have occurred. For example, Beare *et al.* [21] identified 40 breakpoints in pairwise comparisons of four *Coxiella* strains and observed that 75% were within 100 bp of an insertion sequence. Similarly, Darling *et al.* [22] identified 79 inversions in a comparison of eight strains of *Yersinia pestis*, and all of the breakpoints were adjacent to an rRNA operon or within 1500 bp of an insertion sequence. In a third study [16], a comparison of two *Streptococcus mutans* strains revealed a single inversion. However, when 95 additional clinical isolates were examined, numerous other inversions were observed. By contrast, when the K31 chromosome was aligned to that of *C. crescentus* NA1000, more than 60 inversions and 45 large translocations were readily observed (figure 2). This level of genome rearrangements is more than an order of magnitude greater than the examples described above and leads to a well-scrambled genome. Although most inversions flank the origin of replication as observed in the genome comparisons described above, many others do not and instead lead to more local rearrangements.

**Figure 3.**
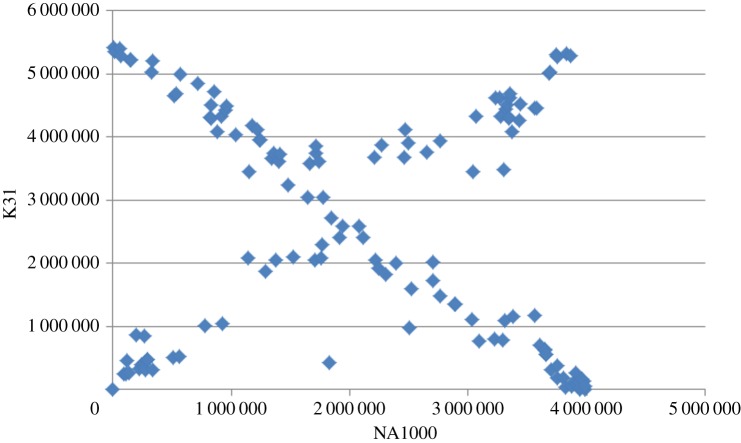
The relative positions of homologous genes in the K31 and the NA1000 chromosomes. The location in each chromosome of the first gene in each homologous block of genes identified in figure 2 was plotted. The origin of replication is located close to position 0 in both genomes.

Several constraints on genome reorganization have been proposed, including a conserved distance from the origin of replication for genes involved in transcription and translation due to copy number differences in fast growing bacteria [23]. To determine whether this phenomenon was limited to fast-growing bacteria, we determined the relative positions of the first gene in each conserved sequence block in the K31 versus NA1000 comparison depicted in figure 2. Despite the extensive rearrangements present in the genome comparison, we found that most genes are in approximately the same place in both genomes or they are in the same place relative to the origin of replication but are on opposite sides of the origin (figure 3). Thus, the position of a gene relative to the origin of replication appears to be conserved in a genus where multiple rounds of replication do not occur, and gene copy number differences do not exceed a factor of two. Furthermore, although it is obvious from figure 2 that the breakpoints of most chromosomal rearrangements do flank the origin of replication, the remainder must be limited to small changes in gene position or to compensating rearrangements to restore the distance between blocks of genes and the origin. Also, as we compared the position of genes in more than 100 conserved sequence blocks, genome position relative to the origin of replication must be important for a relatively large number of genes. One explanation for this phenomenon could be that DNA replication results in hemi-methylated GANTC sites in regions that are responsible for cell-cycle-dependent gene expression. For example, the *ctrA* gene is located a large distance from the origin of replication and has increased expression when hemi-methylated [24]. As CtrA is one of the master cell cycle regulators, this change in CtrA levels is necessary for normal cell growth and progression through the S phase of the cell cycle. Thus, there may be a cascade of effects as chromosome replication progresses and additional gene promoters become hemi-methylated. These results also suggest that rearrangements that impact the distance of critical genes from the origin of replication are not tolerated so that they are lethal events or that they are immediately followed by a second rearrangement that restores the distance from the origin for those genes.

In addition to the rearrangements of major blocks of genes, we also observed eight instances where two or three translation-related genes were in different locations in the two genomes with different flanking genes. Thus numerous small translocations seem to have occurred. One inversion that we examined in detail involved an inversion with breakpoints close to the origin of replication. The inverted segment was asymmetric, with less than 5 kb on one side of the origin and more than 54 kb on the other side. One of the breakpoints was adjacent to the *dnaA* gene so that it is 50 kb closer to the origin in NA1000 than in K31. The other breakpoint was between the genes coding for exodeoxyribonuclease III and the DNA polymerase III epsilon subunit in NA1000 such that the epsilon subunit is 50 kb farther from the origin of replication in K31. The inverted region differs in size by nearly 2 kb in the two species because five separate genes are present in K31 but not in NA1000, and one gene is missing from K31 that is present in NA1000. Thus the inverted region contains at least five small indels that occurred in one of the two genomes.

**Figure 4.**
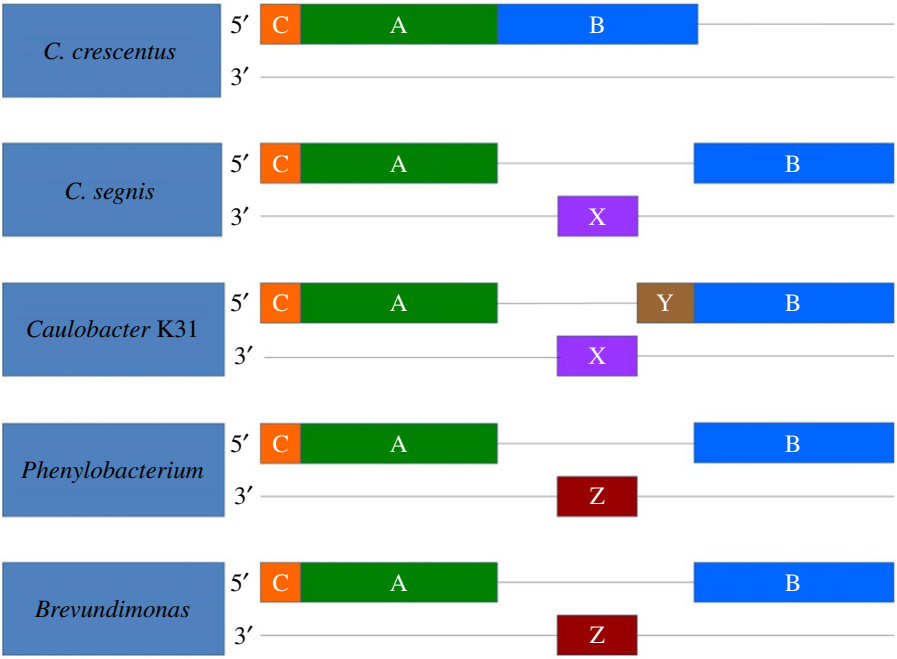
The gene arrangement in the *gatCAB* operon of NA1000, K31 and related bacteria. X, Y and Z represent genes that interrupt the operon that are not present in the NA1000 chromosome.

Another example of the complexity of *Caulobacter* genome rearrangements comes from the three genes for glutamyl-tRNA(Gln) amidotransferase, which comprise a single *gatCAB* operon in NA1000. In K31, the *gatA* and *gatB* genes are expressed from different promoters and are separated by two genes that are not found in the NA1000 genome (figure 4). When other related genomes were compared, *gatA* and *gatB* were separated by one of the two K31 genes (designated X in figure 4) in *C. segnis* TK0059, *C. crescentus* OR37, *Caulobacter* sp. AP07 genomes. However, they are separated by a third gene (designated Z in figure 4) in the chromosomes of two species from closely related genera, *Brevundimonas subvibrioides* and *Phenylobacterium zucineum*. This third gene is found elsewhere in the NA1000 and K31 genomes with a relatively low level of amino acid identity. As K31 and AP07 are on the same branch of the tree, these data suggest that the ancestral *Caulobacter* genome contained gene X. If this hypothesis is true then gene Y must have been inserted into the K31 genome. The single *gatCAB* operon observed in the CB15 genome is found in the CB4 genome as well (D. Scott and B. Ely 2013, unpublished data). As CB4 is closely related to K31, it is likely that gene X has been lost independently in both NA1000 and CB4.

In contrast to the changes described above, it was striking that a conserved 14.5 kb block of 28 genes including 24 ribosomal protein genes remained intact at approximately the same place in the two genomes. This highly conserved block of genes was transcribed in a single direction and had 87% nucleotide identity plus small insertions or deletions that corresponded to approximately 1% of the total region. Part of the reason for this high level of conservation may be that the first 20 ribosomal genes appear to be transcribed as a single operon. Thus, most disruptions of the operon would be lethal due to the failure to express some of these ribosomal protein genes.

Mobile genetic elements are often involved in genome rearrangements [25]. Therefore, we examined the position of the transposase and integrase genes in the NA1000 (*n* = 50) and K31 (*n* = 90) genomes. In the NA1000 and K31 genomes, roughly one-third of the transposases and integrases interrupt regions of homology, another third are located in short non-homologous regions, and the remaining third are associated with a combined insertion and translocation. In most cases, additional genes that are not present in the other genome are adjacent to the transposases. Thus, in addition to mediating gene insertions, transposases may be associated with about one-third of the large translocations observed in the NA1000 and K31 genome comparison. These results indicate that although transposases are involved in some of the genome rearrangements, the majority of the observed rearrangements in the *Caulobacter* genomes must be due to some other mechanism.

## 5. Correlation between genotype and phenotype

With respect to the cell cycle, K31 grown in PYEGGR produces cells that are morphologically similar to those of *C. crescentus*. Both strains attach to surfaces and form biofilms. Comparison of the genes that code for cell cycle/developmental regulators and known components of developmentally regulated structures (flagellum, holdfast, pili and cytoskeletal proteins involved in division) reveals generally high (75–95%) amino acid sequence identity with *C. crescentus*, suggesting that the processes contributing to morphological development have been evolutionarily conserved. For example, the ‘master regulator’ CtrA that controls many key cell cycle events is one of the most highly conserved proteins, with 97% amino acid identity to the corresponding NA1000 protein. The *ctrA* gene nucleotide sequence is also highly conserved (92%), suggesting that there is selection for codon usage as well as for conservation of amino acid sequence. In fact, both the *ctrA* amino acid and nucleotide sequences are highly conserved among the *Alphaproteobacteria*, with greater than 77% identity in both measures for the top 100 matches to the K31 gene in a BLAST search. By contrast, one interesting difference between the genomes is that three of the flagellin genes, *fljMNO*, are contiguous in NA1000, but they are located at three widely separated positions in the K31 genome. In the NA1000 annotation, these three genes are included in three separate mobile elements [4]. If these genes are truly contained in mobile elements, it would provide an explanation for their disparate locations in the K31 genome.

The genome comparison between NA1000 and K31 also revealed the presence of additional copper resistance genes in the genome of K31. K31 has a total of six copper resistance genes in two clusters. One cluster contains copper resistance protein genes CAUL_2631 *copA* and Caul_2630 *copB*, and a similar cluster is found in NA1000. The second contains *copABCD* (CAUL_2346–47 and CAUL_2350–51) with no corresponding region in NA1000. Thus, the K31 genome contains an additional four-gene *copABCD* system that could confer a copper resistance phenotype. To determine whether K31 was actually more resistant to copper inhibition, we performed a series of growth experiments in the presence of a range of copper concentrations. After testing various dilutions of a 60 mg ml^−1^stock solution of CuSO_4_, we discovered that 0.5 mg ml^−1^ was the highest level of copper that allowed K31 growth, and 0.1 mg ml^−1^ was the highest level that allowed NA1000 growth. Thus, K31 is resistant to a fivefold higher concentration of copper than the level that NA1000 tolerates.

As indicated above, experimental observations suggested that K31 grew better in the presence of riboflavin. Riboflavin, also known as vitamin B_2_, is synthesized in many plants and microorganisms, and is used to make FAD and FMN, two key cofactors in many enzymatic reactions. NA1000 contains the five enzymes necessary for the biosynthesis of riboflavin and FAD from GTP. The five enzymes and their various subunits are encoded in five different genes: *ribAB*, *ribD*, *ribH*, *ribE* and *ribF*. Four of these genes, *ribD*, *ribE*, *ribAB* and *ribH*, are encoded in a single operon. This operon seems to be conserved in other closely related *Alphaproteobacteria* such as *Hyphomonas* and *Maricaulis maris*. However, the entire four-gene operon along with nine additional contiguous genes is absent from the K31 genome. The fifth gene, *ribF*, is located 200 kb from the riboflavin operon in *C. crescentus* and codes for a RibF protein that has riboflavin kinase and FAD synthetase activities. It is present in K31 as gene Caul_4052 and codes for a RibF protein that has 84% amino acid identity with the *C. crescentus* protein*.* Another gene annotated as a duplicate *ribH* gene (Caul_1421) is located 500 kb from the riboflavin operon in the *C. crescentus* genome and is also present in K31 as Caul_3045. In *C. crescentus*, the two *ribH* genes code for proteins that have little amino acid sequence similarity, but they are both identified as the beta subunit for riboflavin synthase. However, as K31 does not contain the gene for the alpha subunit, it is unlikely that the K31 RibH enzyme is involved in riboflavin synthesis.

Some freshwater *Caulobacter* isolates have been shown to have properties that are potentially useful for aquatic bioremediation applications, including the degradation of aromatic compounds [26] and the reduction of arsenic [27]. The chlorophenol degradation capacity of *Caulobacter* strain K31 stands out among these. K31 was isolated in an enrichment culture for chlorophenol-tolerant bacteria in groundwater from Karkola, Finland [2]. The groundwater at Karkola percolates through nutrient-poor silt and clay, and is cold (7–8°C), oxygen-deficient, iron-rich and mildly acidic (pH 6–6.5). The aquifer had been contaminated with high levels of chlorophenols for at least two decades by a local sawmill that used polychlorophenol as a fungicide for lumber treatment. Chlorophenols have historically been used as fungicides in agricultural and industrial applications (e.g. wood preservatives applied at lumber mills), and also enter the environment as by-products of paper mills. K31 inhabited groundwater containing up to 190 mg l^−1^ total chlorophenols and was grown in the laboratory on complex media supplemented with 250 mg l^−1^ chlorophenols: 2,4,6-trichlorophenol (TCP), 2,3,4,6-tetrachlorophenol (TeCP) and pentachlorophenol (PCP). In limited testing, K31 was found to degrade TeCP, and to tolerate PCP at relatively high levels, though no evidence was found for degradation of PCP. K31 does not contain the *pcpB* gene (encoding PCP-4-monooxygenase) present in several chlorophenol-degrading *Sphingomonas* isolates from the same site. However, the K31 genome does contain several other genes that code for a variety of monoxygenases. In addition, it has genes for tert butyl ether degradation, alkane metabolism and a tannase/feruloyl esterase that are not found in the NA1000 genome.

In terms of physiology, the K31 genome appears to encode a much larger repertoire of enzymes involved in electron transfer reactions than *C. crescentus*, including more cytochrome *c* variants, respiratory nitrate reductase and many dehydrogenases that are not present in the *C. crescentus* genome. As a groundwater resident growing slowly at low temperatures, K31 probably faces lower dissolved oxygen levels (if not outright anoxia) on a more routine basis than the surface-dwelling bacteria, and may find more respiratory versatility advantageous.

## 6. Conclusion

In summary, K31 is an interesting *Cauolobacter* isolate that has the ability to tolerate copper and chlorophenols, and can grow at consistently low temperatures. The K31 chromosome appears to have been scrambled relative to that of *C. crescentus* NA1000. However, the positions of most genes relative to the distance from the origin of replication are unchanged, indicating that genome rearrangements are constrained. This genome scrambling also makes it difficult to identify individual chromosome rearrangement events. However, it is clear that additional mechanisms must be involved as transposases seem to be associated with only one-third of the observed events. In addition to the genome rearrangements, the K31 chromosome includes numerous insertions and deletions relative to the NA1000 chromosome, so that it contains 1200 more genes, plus an additional 400 genes are present on two very large plasmids. These extra genes provide K31 with increased metabolic versatility.
